# A Population-Based Nationwide Cross-Sectional Study on Preventive Health Services Utilization in Portugal—What Services (and Frequencies) Are Deemed Necessary by Patients?

**DOI:** 10.1371/journal.pone.0081256

**Published:** 2013-11-21

**Authors:** Carlos Martins, Luís F. Azevedo, Orquídea Ribeiro, Luísa Sá, Paulo Santos, Luciana Couto, Altamiro Costa-Pereira, Alberto P. Hespanhol

**Affiliations:** 1 Family Medicine Unit, Social Sciences and Health Department of the Faculty of Medicine of Porto, Porto, Portugal; 2 Centre for Research in Health Technologies and Information Systems (CINTESIS), Information Sciences and Decision on Health Department (CIDES), Faculty of Medicine, University of Porto, Porto, Portugal; The Ohio State University, United States of America

## Abstract

**Background:**

Most of the strategies to induce a more rational use of preventive health services are oriented to the medical side of the doctor-patient relationship. However, the consultation model has changed, and patients now have a more important role in medical consultation. The aim of this study was to assess which healthcare services are deemed necessary, and with what frequency, by adults from the general Portuguese population.

**Methods:**

**Design:** Population-based nationwide cross-sectional study

**Setting:** Portuguese population

**Participants:** One thousand Portuguese adults, surveyed by computer-assisted telephone interviewing and selected by a stratified cluster sampling design.

**Measurements:** Proportions and population prevalence estimates were determined for each healthcare service, taking into account whether respondents considered them necessary, and with what frequency.

**Results:**

Respondent ages ranged between 18 and 97 years, and 520 of 1000 (52%) respondents were women. Among Portuguese adults, 99.2% (95% confidence interval (CI): 98.5 to 99.6) believe that they should undergo general routine blood and urine tests, to be repeated every 12.0 months on average (95% CI: 11.4 to 12.6); 87.4% (95% CI: 85.3 to 89.3) of the respondents reported having actually performed these tests. Of the 15 services surveyed, 14 were considered periodically necessary by more than 60% of respondents. Among the respondents, 37.7% (95% CI: 34.5 to 41.1) reported using healthcare services by their own initiative.

**Conclusions:**

The majority of Portuguese adults believe that they should utilize a great number of healthcare services, on a nearly annual basis; most actually follow this schedule. Our findings indicate a tendency towards the overuse of resources.

Adequate patient-oriented strategies regarding the use of medical tests and preventive interventions—with appropriate information and discussion of risks and harms—are urgently needed, and crucial for achieving a more rational use of healthcare services and for preventing the consequences of over-testing.

## Introduction

In the last few decades, the evolution of the model of medical consultation has changed the way medical decisions are made. From a paternalistic model, to an increasingly shared decision-making trend, the role and the voice of patients has become more present in medical consultation [1]. Another tendency of recent times is the growing importance given to clinical prevention. Advances in technology, with the increasing number of medical tests available for doctors and patients, the cultural belief that more is always better, as well as some disease-mongering strategies, have led preventive medicine to a point where the probability of causing more harm than good is raising great concern [2–4]. A significant number of medical tests are prescribed with a preventive intention to people who are healthy or may have some risk factors. The excessive and unnecessary prescription of medical tests has an important economic impact as well as critical ethical aspects in current clinical practice [4,5].

Previous studies have shown a significant gap between family doctors’ attitudes and scientific evidence related to clinical prevention [6]. Some governments, concerned essentially with the unsustainable increases in health system budgets, have tried to implement some measures to control costs, mainly to reduce that gap. Among these measures, the implementation of pay-per-performance in health systems and the publication of evidence-based guidelines may be considered two strategies most followed by governments. In Portugal, since 2006, the Primary Health Care Reform has implemented a new pay-per-performance system [7,8]. Some of the factors measured in this performance are related to the number of medical tests prescribed by family doctors. In 2011, Portugal has requested financial support from the International Monetary Fund (IMF) and the European Union (EU). As part of the Technical Memorandum of Understanding for the financial support, Portugal committed itself to “establish clear rules for the prescription of drugs and the realization of complementary diagnostic exams (prescription guidelines for physicians) on the basis of international prescription guidelines” and to “continue the publication of clinical guidelines and set in place an auditing system of their implementation” [9]. However, all of these strategies are intended to improve the quality of medical test prescription, based only on the doctor’s action, and ignore the evolution of the medical consultation into a more shared decision-making process, and frequently to a “patient asks, patient gets” decision-making model. In this context, it is vital to know the patients’ beliefs and perceptions about medical tests.

The aim of this study was to assess—in the context of primary care preventive health services—which medical tests are deemed necessary, and with what frequency these services should be utilized, by adults from the general Portuguese population.

## Methods

### Study design

A nationwide cross-sectional study was conducted in a representative sample of the Portuguese adult general population, using computer-assisted telephone interviews (CATI) for data collection.

### Participant selection criteria

The defined target population was the Portuguese adult general population, and the available population included adult individuals living in Portuguese households with a landline telephone (sampling frame). To be eligible, individuals must be adults aged 18 years and over, living in a household (private dwelling) with a landline telephone. Exclusion criteria included the following: having a cognitive or physical disability that hampered the ability to complete a telephone interview; being a nursing home resident or resident in any other type of collective dwelling; and refusal to give informed consent for study participation. 

### Survey sampling methods

To obtain a representative sample of the Portuguese general adult population, a stratified cluster sampling design was used. First, all counties were used as natural strata; in each county, a random sample of households with landline telephone numbers was selected with a probability proportional to the county population size, as estimated by the national census. Next, one eligible resident was randomly selected in each household based on the birthday dates (last birthday in the household was selected). Target quotas were set for age and sex strata in each geographical region, to take into account the likelihood of being available at home for interview and to correct the common overrepresentation in telephone surveys of respondents from the female sex and older age groups [10–12].

A comprehensive set of measures were implemented, which aim to prevent non-response and non-response bias. These included: (1) appropriate selection and specific training of interviewers; (2) inclusion of an introductory presentation as the initial part of household contacts, specifically aimed at capturing participants’ attention, obtaining their informed consent and facilitating participation; and (3) standard operational procedures for contacts and call-backs in case of failed contacts, systematically including eight attempts in different days and at different time-periods of the day. Additionally, in order to correct for sample imbalances and to partially adjust prevalence estimates for selection bias, a set of weighting procedures were implemented [10–12]. Two types of weights were used: (1) weights adapted to the sampling design (stratification and clustering), adjusting for different probabilities of selection among respondents and (2) post-stratification weights, taking into account population distributions by geographical region of residence, gender, and 5-year age categories, based on the Portuguese National Census [13].

### Quality control

The interviewers were experienced and were adequately trained and prepared for the application of the study questionnaire. A first pilot run of 100 interviews was performed to assess the time needed for questionnaire completion and to assess questionnaire language and comprehension issues. A second pilot run was performed during the first 50 interviews. All interviews were supervised by a data collection supervisor; additionally, at least 20% of the interviews were randomly supervised by a study coordinator.

### Sample size

Sample size was determined in order to warrant estimation of proportions with an expected margin of error of 4%, assuming a design effect of 1.5, and an intended confidence level (CI) of 95%. Based on these assumptions, a sample of at least 1,000 adults from the general population was required.

### Instruments and methods for data collection

Data collection was performed between 16th February 2011 and 11th May 2011, using computer-assisted telephone interviews (CATI). A structured questionnaire containing four sections was used: (1) an introductory section presenting study aims and motivation; (2) a section containing questions about the health status of the interviewees; (3) the main research section; (4) and a socio-demographic data collection section.

In the main research section, the interviewees answered three questions regarding the following 15 medical interventions: blood test for cholesterol levels; blood pressure evaluation; blood test for fasting glucose levels; faecal occult blood test; chest X-ray; tetanus vaccine; general routine analysis; thyroid ultrasound; abdominal ultrasound; male-specific interventions of prostate ultrasound and prostate-specific antigen test; and the female specific interventions of mammography, breast ultrasound, gynecological ultrasound, and cervicovaginal cytology. The interviewers were trained to clarify the meaning of each medical test to ensure that participants correctly understood all of the questions.

The three questions were:

- Do you consider that, in your personal case, you should undergo the following medical test, screening or activity?
- If so, how often should you undergo it?
- Do you usually undergo the following medical test, screening or activity?

Question b) was only applied when the answer to question a) was "yes". 

### Statistical analysis

Statistical analysis was performed using the Statistical Package for the Social Sciences Version 19.0 for Windows (SPSS®). All prevalence estimates presented were calculated after taking into account the sampling design and the appropriate weights described above, and using the analysis module Complex Samples of SPSS® 19.0. Point estimates and 95% CI are presented for all prevalence estimates.

Descriptive statistics are presented as absolute frequency (number) and relative frequency (percentage) for categorical variables, and as mean and standard deviation (SD) for continuous variables. Median and inter-quartile range (IQR: 25^th^–75^th^ percentile) were used if the variable empirical distribution function was skewed. When testing hypotheses regarding continuous variables, parametric tests (student’s t test and one factor analysis of variance (ANOVA)) and nonparametric tests (Mann-Whitney and Kruskal-Wallis tests) were used as appropriate, taking into account normality assumptions and the number of groups compared. The Kolmogorov-Smirnov test was used to test normality assumptions of variable distributions. When testing hypotheses regarding categorical variables, Chi-square tests and Fisher’s exact tests were used as appropriate.

Whenever statistical hypothesis testing was used, a significance level of α =5% was considered. 

### Ethical considerations

This study was approved by the São João Health Centre Medical Ethics Committee. Participants provided their verbal informed consent at the beginning of the telephone interview. Written consent was not obtained because interviews were conducted by telephone, without the physical presence of participants. Participants were informed about the estimated duration of the interview and the voluntary character of participation was emphasized. Participants were informed that they could interrupt their participation at any moment of the interview. The interviews were not recorded and participants did not receive any kind of compensation. As a measure to standardize the process of obtaining informed consent, interviewers were specifically trained and were required to read a standardized text. This obtaining of consent procedure was approved by the São João Health Centre Medical Ethics Committee.

## Results

From a total of 2,945 randomly selected households, there were 1,804 with eligible individuals. From the total number of households with eligible individuals, 804 of the selected individuals refused to participate. We obtained 1000 valid interviews, corresponding to a response rate of 55%. The mean duration of the interview was 18 minutes. Respondents were between 18 and 97 years old, and included 520 women, 480 men. In Table 1, we present the general description of sample characteristics.

**Table 1:** Sample characteristics.

**Age (years)**	**% respondents**	**Unweighted Count**
Mean: 45		
Range: 18–97		
From 18 to 29	26.7%	233
From 30 to 39	17.6%	184
From 40 to 49	16.4%	169
From 50 to 59	14.1%	145
From 60 to 69	12.6%	131
From 70 to 79	9.2%	96
80 or more	3.4%	42
**Gender**	Male: 47.8%	480
	Female: 52.2%	520
Geographic distribution (NUTS^*^ II)		
North	35.6%	348
Center	23.3%	230
Lisbon	26.4%	262
Alentejo	7.8%	78
Algarve	3.6%	38
Madeira	1.7%	22
Azores	1.6%	22
**Marital status**		
Single	34.5%	321
Married	56.0%	560
Married but legally separated	0.8%	8
Divorced	2.7%	31
Widowed	6.1%	77
**Highest level of education completed**		
None	2.8%	35
Primary, 1^st^ cycle	23.7%	250
Primary, 2^nd^ cycle	7.0%	72
Primary, 3^rd^ cycle	17.9%	171
Secondary education	23.4%	220
Post-secondary education	3.7%	33
Higher Education, Bachelor	2.1%	21
Higher Education, Graduation	16.6%	167
Higher Education, Masters	2.1%	23
Higher Education, PhD	0.6%	6
**Professional occupation**		
Has a profession	53.3%	534
Student	11.7%	96
Homemaker	7.0%	74
Retired	21.8%	234
Unemployed	6.3%	60
**Residence location**		
Urban	55.3%	559
Rural	44.7%	441

* NUTS: Nomenclature of Territorial Units for Statistics

Table 2 shows that 58.9% of the interviewees considered themselves to have a good to excellent health status. The self-reported prevalence in our sample was 25.0% for hypertension, 24.3% for hypercholesterolemia, 6.6% for diabetes, 10.4% for heart problems, 9.6% for asthma and/or COPD, 13.6% for depression, and 3.0% for cancer.

**Table 2:** Self-perceived health status, medical conditions and risk factors.

	**% respondents**	**Unweighted Count**
**Health status**		
In general, would you say your health is:		
Excellent	10.9%	107
Very good	15.3%	139
Good	32.7%	326
Fair	30.4%	314
Poor	10.8%	114
**Medical conditions and risk factors**		
Hypertension	25.0%	246
Elevated cholesterol	24.3%	270
Diabetes	6.6%	76
Heart problems	10.4%	109
Osteomuscular pain	58.6%	585
Asthma and/or COPD*	9.6%	101
Gastritis or peptic ulcer disease	11.2%	122
Anxiety	37.9%	378
Depression	13.6%	141
Overweight or obesity	26.0%	260
Smoker	17.3%	183
Cancer	3.0%	34
I am healthy, I don’t have any disease	15.9%	158

* COPD: chronic obstructive pulmonary disease

Table 3 shows the prevalence of individuals that believe they should undergo a specific medical activity, the mean time of frequency, and the prevalence of those that undergo the activity. There is an estimated prevalence of 99.2% (95% CI: 98.5 to 99.6) of Portuguese adults that believe they should undergo general routine blood and urine tests, with a mean frequency interval of 12.0 months (95% CI: 11.4 to 12.6), and 87.4% (CI: 85.3 to 89.3) report that they usually undergo this activity. 

**Table 3:** Prevalence of individuals believing the proposed health services to be necessary, with optimal respective frequencies.

	**Yes, I believe I should undergo this medical test/activity**				**How often?**			**Yes, I utilize this medical test/activity**			
			**95% CI**			**95% CI**				**95% CI**	
**Health services**	**n**	**(%)**	**Lower**	**Upper**	**Mean time (months)**	**Lower**	**Upper**	**n**	**(%)**	**Lower**	**Upper**
**Routine blood & urine tests**	983	99.20%	98.50%	99.60%	12.0	11.4	12.6	875	87.40%	85.30%	89.30%
**Tetanus vaccine**	883	93.90%	92.30%	95.30%	109.1	107.0	111.2	820	85.10%	82.90%	87.10%
**Cholesterol evaluation**	921	93.20%	91.30%	94.60%	10.3	9.9	10.7	806	79.50%	76.80%	81.90%
**Evaluation of blood pressure**	869	87.60%	85.30%	89.50%	3.3	3.0	3.7	798	78.40%	75.90%	80.80%
**Evaluation of glucose**	839	86.50%	84.20%	88.50%	9.9	9.5	10.3	699	68.80%	65.70%	71.80%
**Cervicovaginal cytology**	404	83.90%	80.70%	86.60%	14.4	13.6	15.1	338	65.30%	61.00%	69.40%
**Gynecological ultrasound**	388	81.40%	77.90%	84.50%	13.5	13.1	14.0	310	61.10%	56.70%	65.20%
**Breast ultrasound**	386	77.70%	73.90%	81.10%	14.7	14.0	15.4	315	59.10%	54.10%	64.00%
**Mammography**	397	77.40%	73.50%	80.90%	15.4	14.6	16.1	317	60.20%	55.60%	64.70%
**Lung X-ray**	665	70.00%	66.70%	73.00%	15.5	14.7	16.3	435	44.00%	40.70%	47.50%
Evaluation of PSA*	294	67.30%	62.80%	71.50%	14.7	13.1	16.4	170	33.90%	29.80%	38.40%
**Abdominal ultrasound**	615	67.20%	64.10%	70.10%	15.4	14.3	16.6	293	29.00%	26.10%	32.10%
FOBT**	557	65.30%	62.10%	68.40%	13.7	12.8	14.6	163	16.70%	14.20%	19.40%
**Prostate ultrasound**	274	61.50%	56.90%	65.80%	16.1	14.5	17.8	142	28.50%	24.60%	32.80%
**Thyroid ultrasound**	385	46.40%	42.90%	49.90%	16.3	14.8	17.8	164	17.20%	14.50%	20.30%

* PSA: prostate specific antigen; **FOBT: fecal occult blood test

There is an estimated prevalence of 93.2% (95% CI: 91.3 to 94.6) of Portuguese adults that believe they should undergo cholesterol evaluation; 87.6% (95% CI: 85.3 to 89.5), blood pressure evaluation; and 86.5% (95% CI: 84.2 to 88.5), glucose evaluation. Among Portuguese adult women, 83.9% (80.7 to 86.6) consider cervicovaginal cytology necessary; 81.4% (95% CI 77.9 to 84.5), a gynecological ultrasound; 77.7% (95% CI: 73.9 to 81.1), a breast ultrasound; and 77.4% (95% CI: 73.5 to 80.9), mammography. Among Portuguese adult men, 67.3% (95% CI: 62.8 to 71.5) consider a PSA test necessary and 61.5% (95% CI: 56.9 to 65.8) consider a prostate ultrasound necessary.

For 14 of the 15 medical activities conidered, there was an estimated prevalence of more than 60% of Portuguese adults believing that they should undergo that specific medical activity. 

Regarding the optimal frequency identified by Portuguese adults for each medical test or activity, blood pressure evaluation, glucose measurements, and cholesterol measurements were considered necessary with the shortest mean intervals: 3.3 months (95% CI: 3.0 to 3.7), 9.9 months (95% CI: 9.5 to 10.3) and 10.3 months (95% CI: 9.9 to 10.7), respectively. The mean optimal frequency was between 12 and 15.5 months for 9 of the 15 medical activities considered.

In Table 4, we have made a depuration: for each specific medical test, the respondents with declared conditions or risk factors that would justify the performance of that medical test were withdrawn from the analysis By analyzing the data of those not requiring the tests, we verify that only for glucose evaluation does a statistically significant difference exist: the estimated prevalence of individuals that consider this test necessary is lower, at 76.3% (95% CI: 69.3 to 82.1). In the other medical tests, the results are quite similar to the global group analysis. 

**Table 4:** Prevalence of individuals without risk factors* believing the proposed health services to be necessary, with optimal respective frequencies.

	**Yes, I believe I should undergo this medical test/activity**				**How often?**			**Yes, I utilize this medical test/activity**			
			**95% CI**			**95% CI**				**95% CI**	
**Health services**	**n**	**(%)**	**Lower**	**Upper**	**Mean time (months)**	**Lower**	**Upper**	**n**	**(%)**	**Lower**	**Upper**
**Cholesterol evaluation**	194	89.90%	85.30%	93.20%	11.67	10.95	12.39	165	72.90%	66.30%	78.60%
**Evaluation of blood pressure**	221	85.10%	80.10%	89.00%	4.58	3.75	5.41	201	74.60%	68.90%	79.50%
**Evaluation of glucose**	147	76.30%	69.30%	82.10%	10.08	9.06	11.11	110	55.30%	47.90%	62.40%
**Cervicovaginal cytology**	385	84.20%	81.00%	87.00%	14.41	13.63	15.2	321	65.50%	61.00%	69.60%
**Gynecological ultrasound**	310	80.60%	76.50%	84.20%	13.58	13.08	14.09	247	60.00%	55.20%	64.70%
**Breast ultrasound**	322	77.00%	72.80%	80.70%	14.89	14.08	15.7	265	59.30%	53.70%	64.60%
**Mammography**	331	76.60%	72.30%	80.30%	15.39	14.49	16.28	261	58.80%	53.70%	63.70%
**Lung X-ray**	549	67.80%	64.30%	71.20%	15.55	14.61	16.49	343	40.90%	37.20%	44.60%
Evaluation of PSA**	281	66.90%	62.30%	71.30%	14.96	13.25	16.67	160	33.00%	28.80%	37.40%
**Abdominal ultrasound**	587	67.30%	64.20%	70.30%	15.23	14.09	16.38	280	29.20%	26.30%	32.40%
FOBT***	514	66.90%	63.50%	70.10%	13.76	12.77	14.75	153	17.10%	14.50%	20.10%
**Prostate ultrasound**	263	61.30%	56.60%	65.80%	16.3	14.61	17.99	134	27.80%	23.90%	32.00%
**Thyroid ultrasound**	379	46.50%	43.00%	50.00%	16.24	14.69	17.78	159	17.00%	14.30%	20.10%

* For cholesterol evaluation, patients with high cholesterol, diabetes, heart problems, a family history of heart problems, smoking habits, hypertension, or obesity (BMI ≥30) were excluded. For evaluation of blood pressure, patients with diabetes, heart problems, a family history of heart problems, smoking habits, hypertension, or obesity (BMI ≥30) were excluded. For evaluation of glucose, overweight (BMI ≥25) or obese patients, and patients with diabetes, hypertension, a family history of diabetes, or high cholesterol were excluded. For cervicovaginal cytology, patients with a personal or family history of cancer of the cervix, or simply "uterus", were excluded. For gynecological ultrasound, patients with a personal or family history of ovarian, breast, uterine, or vulvae cancers were excluded. For breast ultrasound and mammography, patients with a personal or family history of breast or ovarian cancer were excluded. For lung X-ray, patients with a personal or family history of lung cancer were excluded. For evaluation of PSA** and prostate ultrasound, patients with a personal or family history of prostate cancer were excluded. For faecal occult blood test (FOBT***), patients with a personal or family history of colon or rectal cancer were excluded. For abdominal ultrasound, patients with a personal or family history of liver or pancreatic cancer were excluded. For thyroid ultrasound, patients with a personal or family history of thyroid cancer were excluded.

** PSA: prostate specific antigen; ***FOBT: fecal occult blood test

**Figure 1:**
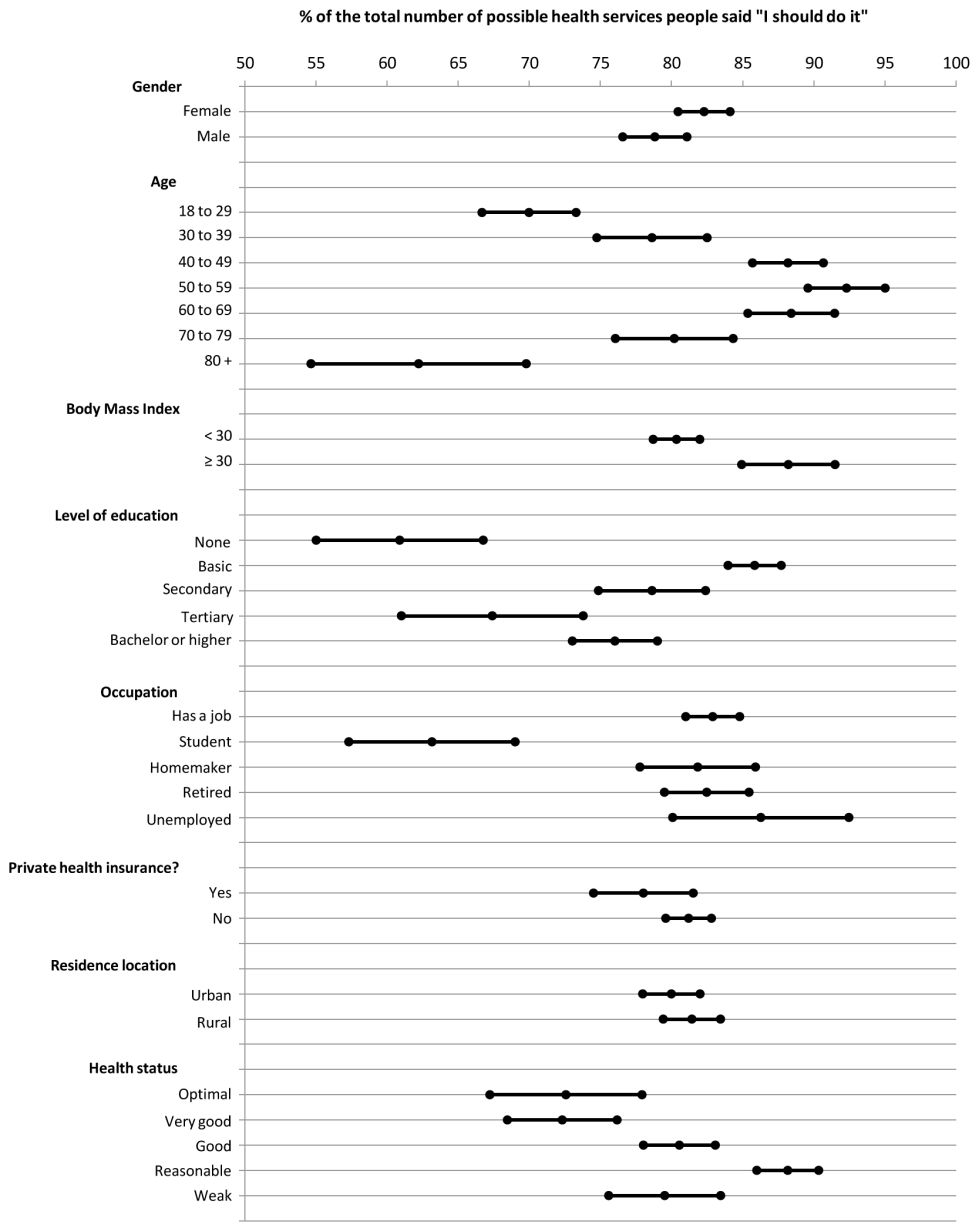
Factors influencing the total number of health services considered necessary by respondents.

In Figure 1, we observe the association of some factors with the percentage of the total number of medical tests/interventions that our sample's respondents deem necessary. Since the total number of possible medical tests/interventions considered in the questionnaire was different between men and women, we have calculated the percentage of the total number that the respondents considered necessary. There were no statistically significant differences between male and female respondents. The 40 to 69-year age group reported a significantly higher number of tests/interventions than the younger age groups (p <0.001). Respondents with a Body Mass Index (BMI) ≥30 also reported a significantly higher number of necessary tests/interventions (p <0.001). Respondents with a basic level of education reported a significantly higher number of tests/interventions. Students reported a significantly lower number of tests/interventions compared to respondents of other occupations. There were no statistically significant differences between respondents having private health insurance and those that did not, nor between urban and rural respondents. Finally, regarding the self-reported health status, respondents claiming a reasonable health status reported a significantly higher number of tests/interventions than those claiming a good to optimal health status.

Table 5 shows that a significantly higher proportion of the Portuguese adults (37.7%; 95% CI: 34.5 to 41.1) chooses to undergo medical tests through their own initiative, rather than through their doctors’ initiative or by mutual agreement with their doctors. Only 3.8% (95% CI: 2.8 to 5.0) report never undergoing medical tests.

**Table 5:** The statement that best describes a respondent’s situation when undergoing medical tests.

	**Estimate**	**95% Confidence Interval**		**Unweighted**
		**Lower**	**Upper**	**Count**
**You undergo medical tests by your own initiative (you ask your doctor to prescribe them)**	37.7%	34.5%	41.1%	369
**You undergo medical tests by your doctor**’s **initiative** (**your doctors recommends that you undergo the tests**)	30.3%	27.5%	33.2%	311
**You undergo medical tests** by **mutual agreement between you and your doctor**	28.2%	25.3%	31.3%	282
**You don’t undergo medical tests**	3.8%	2.8%	5.0%	38

## Discussion

These results show that the vast majority of the adult Portuguese population considers a great number of medical tests necessary on a nearly annual basis, and that most follow these testing parameters. A more robust example of this statement is the result obtained for “routine blood and urine tests”. When discussing periodic health examinations or routine health checks, a popular expression in Portuguese language, ‘análises gerais’—which could be literally translated into English as ‘general analysis’—always comes to Portuguese patients’ minds. It is a frequent reason for consultation and a frequent request in a Portuguese general practice consultation: “Doctor, I want to do a ‘general analysis’”. A fixed panel of tests is not included in these ‘general analysis’, but usually includes a urinalysis and blood tests for complete blood count, glucose, total and HDL cholesterol, triglycerides, hepatic enzymes, and creatinine. The patient’s perceived need for yearly routine blood and urine tests may be linked to the traditional concept of the yearly periodic health examination and seems to be strongly and culturally rooted in Portuguese population. In Portugal, there are no official recommendations for the frequency of adult periodic health examinations, nor for routine blood tests. The Portuguese Ministry of Health recommends the following three cancer screenings: breast cancer screening by mammography every 2 years, for women from 50 to 69 years old; colo-rectal cancer screening by faecal occult blood test every 1–2 years, for adults from 50 to 74 years old; and cervical cancer screening with cervicovaginal cytology for women between 25 and 60 years, every 3 years after 2 annual normal tests [14].

Nowadays, we have scientific evidence that general health checks in adults do not reduce overall or specific (e.g., cardiovascular or cancer causes) morbidity or mortality, although they increase the number of new diagnoses [15]. Previous studies in other countries have shown a trend in patients to overestimate the benefits achieved by screening and preventive treatments [16–19]. Other studies have also shown a trend in patients to undergo some tests, e.g., cancer screening tests, more often, and in younger ages more often than scientific evidence-based recommends [20]. 

Our results also show that patients’ perception of required medical testing is far from what scientific evidence recommends. Our patients do not show a capacity for discriminating between medical tests that are performed on a usual and evidence-based recommendation, and those that are not. For example, there is no statistically significant difference between the estimated prevalence of women who consider cervicovaginal cytology necessary and those who consider a gynecological ultrasound necessary; the same is true for breast ultrasound and mammography. Another major incoherence is the observation that more Portuguese adults consider a lung X-Ray necessary than a fecal occult blood test.

Except for the tetanus vaccine, the reported mean optimal frequencies for each medical test or activity are far more regular than usually recommended. This is true even for the medical tests usually recommended for target groups of certain ages.

It is interesting to observe that factors like gender, private health insurance status, and residence location (urban or rural), do not influence the total number of health services that patients consider necessary. However, obesity (BMI ≥30), having only the basic level of education, or belonging to the 40 to 69-year age group seem to be related to a higher number of health services that patients consider necessary. To observe whether these findings are replicated in other countries and to study what justifies these associations would be interesting.

Taking into account all of these facts, and also that a significantly higher proportion of Portuguese adults consider undergoing medical tests more often by their own initiative, we may conclude that strategies aiming for a more evidence-based and rational prescription of medical tests should be patient-oriented. Also, we raise the hypothesis that patient-oriented strategies may be more successful in achieving this goal than doctor-oriented strategies, as observed thus far. It is important to clarify that when Portuguese patients report that they undergo medical tests more by their own initiative, this does not mean that they will undergo the tests without a medical prescription. For most of the Portuguese population, going directly to the laboratory for medical testing without a medical prescription is financially unsustainable. The patients are reporting that they feel confident in getting the prescription they want from their doctors. 

Doctor-oriented strategies, like financial incentives and pay-per-performance systems, to induce more rational prescribing of medical tests do not take into account the recent evolution of the medical consultation and the way decisions are made in the consultation. Although doctor-oriented strategies may have some positive aspects, these may be a disturbing factor for the doctor-patient relationship, and should always be complemented with patient-oriented strategies. This is not only a matter of saving costs, it is also a matter of “primum non nocere”, a matter of avoiding harm associated with medical tests, including unnecessary diagnoses that lead to unnecessary treatments and false alarms, which often carry secondary effects and/or psychological distress.

Our study has some limitations. First, we obtained a 55% response rate, which may be considered as low. Low response rates are frequently found as a limitation in this type of population survey and may constitute a source of selection bias. Changes in telecommunications, marketing, and culture are some of the factors that are thought to contribute to the growing threat of non-response to household telephone surveys [10,21]. 

Second, when a participant was asked whether he or she considered a medical test/intervention necessary, we did not clarify whether this was in a preventive or a diagnostic setting. We believe it would have been difficult for most of the individuals to differentiate between these two concepts; therefore, to clarify the intentions of participants is ultimately not possible. However, that the primary care preventive services were the main setting of the study was generally clear. 

Third, when we analyse the table 4 results, we have to take in consideration that we are dealing with a self perceived assessment of the participant's medical condition that may not correspond to the true need of a health service.

Fourth, the questionnaire focused on a limited set of medical tests/interventions. To include other medical tests—for example, more serum tumor markers or computer tomographies—would have been interesting. We did not include these tests because doing so would have excessively extended the duration of the interviews.

Fifth, in order to select a representative sample of the Portuguese adult population, we implemented a stratified cluster sampling of households and randomly selected participants in each household based on birth dates. However, we implemented quotas for age and gender strata for each geographical region. Thus, we have some inherent limitations of the quota-sampling scheme.

Further research is needed to investigate which methods could be more cost-effective in patient-oriented strategies, which aim to clarify the meaning of scientific evidence, as well as the costs and the risks/harms of medical tests, for the patients. In this context, the need for further research regarding the harms of general health checks is required. Also, to verify if these results are also found in other countries, as well as further studies regarding patients’ views and knowledge about the risks and harms of medical tests, would be interesting.

Despite the limitations of our study, we believe that these results are a reflection of the evolution of medical consultation in Western culture and trends in population demand for health care. The results presented are certainly generalizable to the vast majority of Western European countries and many other developed/developing countries in the world. The widespread nature of the problem, the importance of this issue for the health of individuals and for the sustainability of health systems makes its discussion more urgent and necessary than ever. The development of educational interventions aiming to inform populations about the real impact and adequacy of certain heathcare services is crucial for prevention of their inadequate demand and to promote the implementation of preventive services that may bring benefit and a positive impact to each patient’s health.
